# The effect of HIV-associated tuberculosis, tuberculosis-IRIS and prednisone on lung function

**DOI:** 10.1183/13993003.01692-2019

**Published:** 2020-03-12

**Authors:** Cari Stek, Brian Allwood, Elsa Du Bruyn, Jozefien Buyze, Charlotte Schutz, Friedrich Thienemann, Adele Lombard, Robert J. Wilkinson, Graeme Meintjes, Lutgarde Lynen

**Affiliations:** 1 Dept of Clinical Sciences, Institute of Tropical Medicine, Antwerp, Belgium; 2 Wellcome Centre for Infectious Diseases Research in Africa, Institute of Infectious Disease and Molecular Medicine, University of Cape Town, Cape Town, South Africa; 3 Dept of Medicine, University of Cape Town, Cape Town, South Africa; 4 Division of Pulmonology, Dept of Medicine, Stellenbosch University, Stellenbosch, South Africa; 5 Dept of Medicine, Imperial College London, London, UK; 6 The Francis Crick Institute, London, UK

## Abstract

Residual pulmonary impairment is common after treatment for tuberculosis (TB). Lung function data in patients with HIV-associated TB are scarce, especially in the context of paradoxical TB-associated immune reconstitution inflammatory syndrome (TB-IRIS) and prophylactic prednisone. We aimed to determine the prevalence of lung function abnormalities in patients with HIV-associated TB and CD4 counts ≤100 cells·μL^−1^ and assess the effect of prophylactic prednisone and the development of paradoxical TB-IRIS on pulmonary impairment.

We performed spirometry, 6-min walk test (6MWT) and chest radiography at baseline (week 0) and at weeks 4, 12 and 28 in participants of the PredART trial, which evaluated a 28-day course of prednisone to prevent TB-IRIS in patients with HIV-associated TB commencing antiretroviral therapy.

153 participants underwent spirometry and/or 6MWT at one or more time points. Abnormal spirometry measurements were present in 66% of participants at week 0 and 50% at week 28; low forced vital capacity was the commonest abnormality. Chest radiographs showed little or no abnormalities in the majority of participants. Prednisone use resulted in a 42 m greater 6-min walk distance and a 4.9% higher percentage of predicted forced expiratory volume in 1 s at week 4; these differences were no longer significantly different from week 12 onwards. TB-IRIS did not significantly impair lung function outcome.

Residual pulmonary impairment is common in HIV-associated TB. In patients with low CD4 counts, neither prophylactic prednisone as used in our study nor the development of TB-IRIS significantly affected week-28 pulmonary outcome.

## Abstract 2

**Post-tuberculosis lung disease is common in patients with HIV-associated TB at high risk of TB-IRIS (CD4 count ≤100 cells·μL^−1^). Neither TB-IRIS itself, nor prednisone treatment, affected long-term pulmonary outcomes in a South African clinical setting.** http://bit.ly/2RjMl9c

## Introduction

Tuberculosis (TB) is frequently complicated by lung function impairment. The odds of abnormal spirometric test results are two- to three-fold higher in patients with a history of TB compared to those without a history of TB, with both obstructive and/or restrictive impairments occuring [1, 2]. Studies of lung function in people with a history of TB found abnormal lung function in 45–87% of subjects [3–5], with even higher proportions reported in multidrug-resistant TB [6, 7]. HIV is an independent risk factor for predominantly obstructive lung function impairment [8, 9]; however, data on lung function impairment in HIV-associated TB are scarce, as HIV co-infected patients are frequently excluded from studies. TB-related lung damage may be less common in those co-infected with HIV; however, current data are conflicting. 1) A single large study found less pulmonary impairment after TB in patients who were HIV-positive [4], while other studies have not supported this beneficial association between HIV status and spirometric outcomes [5, 10, 11]; 2) furthermore, chest radiography findings in patients with HIV-associated TB and low CD4 counts (CD4 <200 cells·μL^−1^) are frequently normal or atypical [12]; and 3) HIV co-infection results in lower levels of several of mediators usually implicated in inflammatory lung damage [13]. Conversely, paradoxical TB immune reconstitution inflammatory syndrome (TB-IRIS) after the start of antiretroviral therapy (ART) causes inflammation and high levels of inflammatory mediators [14–17]. Two recent studies suggested that TB-IRIS may cause lung function impairment [18, 19]; one showing increased inflammation, as assessed by positron emission tomography (PET)-computed tomography (CT) scan, was associated with worse lung function outcomes [18]. However, in these cohorts only small numbers of patients developed predefined TB-IRIS, yet the authors hypothesise that increases in pulmonary inflammation can occur as part of not clinically recognised TB-IRIS. To date, the only other study exploring the relationship between TB-IRIS and lung function found worse spirometric outcomes in the three patients who developed TB-IRIS compared to 11 controls [15].

Corticosteroids reduce inflammation and inhibit several of the immune mediators implicated in lung damage during TB [20–23], and may therefore reduce lung function impairment associated with TB. Previous studies assessing this association did not find a significant effect of corticosteroid use on tests of lung function [24–27]. However, most of these studies were performed before the introduction of rifampicin, and none included HIV co-infected patients.

In this study, we determined the prevalence of lung function impairment over time in a randomised controlled trial of patients treated for HIV-associated TB. Additionally, we assessed the effect of prednisone evaluated in comparison to placebo for prevention of TB-IRIS on pulmonary outcome in this patient group.

## Methods

### Design and setting

This was a substudy of the PredART trial [28], a randomised, double-blind, placebo-controlled trial assessing the efficacy of prednisone to prevent TB-IRIS. Participants were recruited from Khayelitsha, a peri-urban township in Cape Town, South Africa. They were ambulant and treated in an outpatient setting. They received either prednisone or placebo (40 mg·day^−1^ for 2 weeks, followed by 20 mg·day^−1^ for 2 weeks), starting within 48 h of starting ART. The study drug could be replaced with open-label prednisone (1.5 mg·kg^−1^·day^−1^ for 2 weeks, followed by 0.75 mg·kg^−1^·day^−1^ for 2 weeks, or longer if clinically indicated) for treatment of TB-IRIS. TB-IRIS events were adjudicated by three clinical experts using the International Network for the Study of HIV-associated IRIS consensus case definition [29].

Enrolment for the substudy started later than the main trial. Participants already enrolled in the main trial could enrol in the substudy from their next eligible visit, if they had not completed the main trial yet. Participants treated for multidrug-resistant TB or who prematurely discontinued TB treatment were excluded from the analysis at week 28. Successful completion of TB treatment was defined following South African National Tuberculosis Management Guidelines [30].

The substudy was approved by the same ethical committees that approved the main trial [28]. Separate written informed consent was obtained.

### Procedures

Substudy visits were scheduled at weeks 0 (initiation of ART and study drug), 4, 12 and 28. At each visit, pulmonary symptoms (cough, dyspnoea at exertion and dyspnoea at rest) were assessed by the trial doctor, and spirometry and 6-min walk tests (6MWT) were performed. Forced vital capacity (FVC) and forced expiratory volume in 1 s (FEV_1_) were measured using a desktop spirometer (Spirolab III; MIR, Rome, Italy). Tests were performed and results interpreted using the 2005 American Thoracic Society/European Respiratory Society guidelines for spirometry, using National Health and Nutrition Examination Survey (NHANES) reference ranges [31]. We defined four possible outcomes (supplementary figure S1): normal lung function (FEV_1_/FVC ≥70% predicted and FVC ≥80% pred), low FVC (FEV_1_/FVC ≥70% pred and FVC <80% pred), obstructive impairment with and without low FVC (FEV_1_/FVC <70% pred) and “technically incorrect”, comprising participants who performed spirometry, but did not meet criteria for interpretation (supplementary table S1). 6MWTs were performed following the 2002 American Thoracic Society guidelines [32] on a 20-m outdoor track. Chest radiography was performed at weeks 0 and 28. After completion of the study, digitised chest radiographs were scored by two independent blinded readers, using an adapted version of the Timika score as described by Kriel *et al.* [33]. Where discrepancy existed, defined as a difference in score of >10 points, a third reader evaluated the chest radiograph and consensus was found using a two-to-one vote.

### Statistical methods

Analyses were performed using Stata 15.1 (StataCorp, College Station, TX, USA). For categorical variables and continuous variables, proportions and medians with interquartile ranges (IQR), respectively, were estimated at different time points. Comparison of the Karnofsky Performance Score (KPS) between groups was done using a mixed-effects proportional odds model. Correlation between chest radiograph score and lung function was done using mixed-effects regression (linear or logistic) models. Mixed-effect models including a random intercept and covariates were used to model the evolution of pulmonary function over time. The effect of prophylactic prednisone on pulmonary function was tested using a test of the interaction of prednisone and visit number; participants receiving prednisone as treatment for TB-IRIS were analysed in their intention-to-treat arm (*i.e*. study placebo or prednisone). The effect of TB-IRIS was tested using a joint test of the main effect of TB-IRIS and its interaction with visit number. A p-value <0.05 was considered statistically significant.

## Results

**FIGURE 1:**
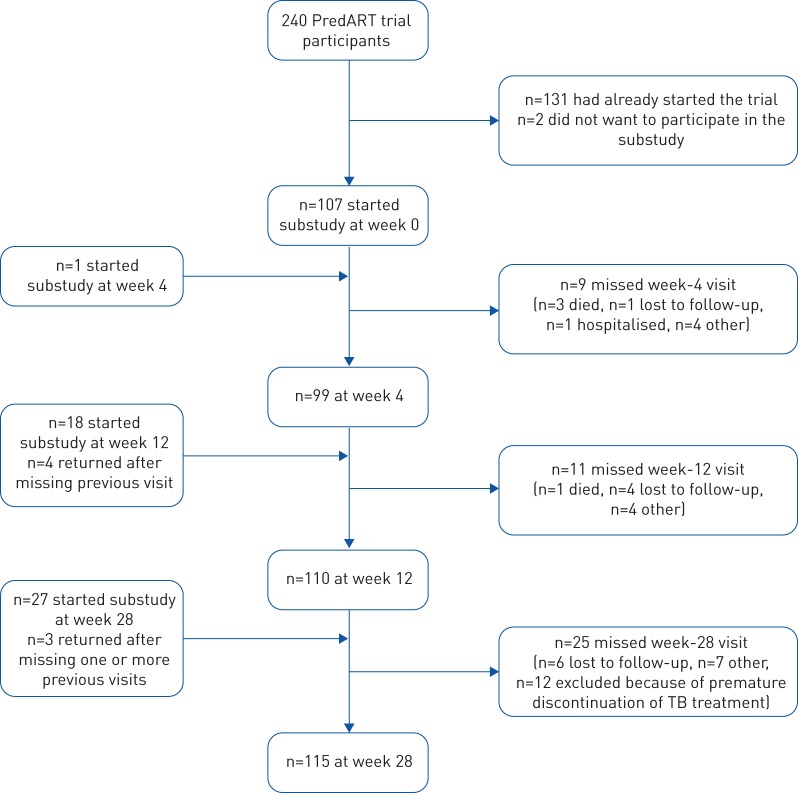
Number of participants per visit.

Between January 2015 and February 2016, 153 participants were enrolled, 77 from the prednisone arm and 76 from the placebo arm. The flow of participants is described in figure 1; baseline characteristics are summarised in table 1. 71 (46%) participants developed TB-IRIS: 30 in the prednisone and 41 in the placebo arm; 46 (30%) participants received open-label prednisone as treatment for TB-IRIS: 16 in the prednisone and 30 in the placebo arm.

**TABLE 1:** Baseline characteristics and participants developing tuberculosis-associated immune reconstitution inflammatory syndrome (TB-IRIS) and receiving prednisone as treatment according to trial arm

	**Prednisone**	**Placebo**
**Male**	49 (64)	41 (54)
**Age years**	38 (31–43)	38 (31–44)
**CD4 count cells·μL^−1^**	46 (24–81)	50 (24–86)
**HIV viral load log_10_ copies·mL^−1^**	5.5 (5.2–5.8)	5.6 (5.3–5.9)
**Extrapulmonary TB**^#^	24 (38)	33 (55)
**Microbiologically confirmed TB**	53 (70)	59 (79)
**Previous TB**	6 (8)	8 (11)
**Time on TB treatment at week 0 days**	16 (15–22)	16 (14–21)
**Smoking status at week 0**^¶^ **pack-years**		
Never smoked	36 (55)	41 (67)
Ever smoked	30 (45)/2.2 (0.8–5.0)	20 (33)/3.75 (1.0–10.0)
**Previous lung disease**	1 (1.3)	0 (0)
**Spirometry**		
FEV_1_ % pred	75 (61–88)	73 (59–86)
FVC % pred	74 (66–89)	73 (65–81)
FVC/FEV_1_ %	83 (79–85)	82 (77–86)
**TB-IRIS**	30 (39)	41 (54)
**Treatment with open-label prednisone for suspected TB-IRIS**	16 (21)	30 (39)

Data are presented as n (%) or median (interquartile range). TB: tuberculosis (pulmonary TB: participants with one or more pulmonary signs or symptoms (such as cough, shortness of breath, abnormal chest radiograph) of TB at presentation; extrapulmonary TB: participants with signs of extrapulmonary tuberculosis (such as pleural effusion or enlarged lymph nodes); microbiologically confirmed TB: participants with *Mycobacterium tuberculosis* detected on culture, with the use of the Xpert MTB/RIF assay (Cepheid, Sunnyvale, CA, USA), or as positive acid-fast bacilli on smear microscopy); FEV_1_: forced expiratory volume in 1 s; FVC: forced vital capacity. ^#^: n=123; ^¶^: n=127.

### Overall prevalence of lung function abnormalities

#### Symptoms

80% of participants reported cough, dyspnoea on exertion and/or dyspnoea at rest as one of their presenting TB symptoms. At week 0, a median 16 (IQR 15–21) days into TB treatment, 50% had one or more of these symptoms. Symptoms improved over time; however, 8% of participants who successfully completed their TB treatment at week 28 still had one or more respiratory symptoms (table 2).

**TABLE 2:** Symptoms, spirometric outcomes, 6-min walk distance (6MWD) and chest radiograph scores in the whole study group at different time points

	**Week 0**^#^	**Week 4**	**Week 12**	**Week 28**
**Symptoms**				
Participants	107	99	110	111
Cough	40 (37.4)	29 (29.3)	14 (12.7)	5 (4.5)
Dyspnoea at exertion	37 (34.6)	27 (27.3)	12 (10.9)	5 (4.5)
Dyspnoea at rest	11 (10.3)	8 (8.1)	3 (2.7)	1 (0.9)
Total^¶^	54 (50.5)	43 (43.4)	20 (18.2)	9 (8.1)
**Spirometry outcome**				
Participants	106	96	110	114
Normal^+^	28 (26.4)	36 (37.5)	50 (45.5)	54 (47.4)
Low FVC^§^	48 (45.3)	42 (43.8)	43 (39.1)	44 (38.6)
Obstruction with or without low FVC^ƒ^	7 (6.6)	7 (7.3)	10 (9.1)	11 (9.7)
Technically incorrect^##^	23 (21.7)	11 (11.5)	7 (6.4)	5 (4.4)
**6MWD**				
Participants	102	91	104	113
6MWD m	520 (465–576)	524 (450–579)	539 (483–608)	585 (520–655)
**Chest radiography**				
Participants	135			61
Chest radiograph score	4 (0.8–11.7)			0.9 (0–3.75)

Data are presented as n, n (%) or median (interquartile range). FVC: forced vital capacity. ^#^: the start day of antiretroviral therapy and prednisone/placebo (median 16 days after start of anti-tuberculosis treatment); ^¶^: cough and/or dyspnoea at exertion and/or dyspnoea at rest; ^+^: forced expiratory volume in 1 s (FEV_1_)/FVC ≥70% predicted and FVC ≥80% pred; ^§^: FEV_1_/FVC ≥70% pred and FVC <80% pred; ^ƒ^: FEV_1_/FVC <70% pred; ^##^: test not fulfilling criteria for interpretation.

#### Spirometry

426 spirometry tests were performed. In 15 substudy participants, spirometry was not performed at one or more time points; five of these participants were too ill to perform spirometry, the remaining 10 were not undertaken for logistic reasons. Spirometic outcomes at different time points are shown in table 2. The proportion of participants with a normal spirometry outcome was 26.4% at week 0, increasing to 47.4% at week 28. Low FVC was the commonest abnormality. The proportion of participants with obstruction with or without low FVC was low and roughly the same over time. We found no effect of CD4 cell count recovery on change over time of spirometric outcomes (p=0.71 for FEV_1_).

At week 0, 23 (21.7%) participants performed a technically incorrect test; the main reason was the inability to exhale for 6 s. To assess if this possibly reflected more impaired lung function, we compared 6-min walk distance (6MWD) and KPS between participants with correctly and incorrectly performed tests. On average, the participants with an incorrect test had a 6MWD 56 m (95% CI 30–83 m, p<0.001) shorter and a lower KPS (p<0.001). At week 12, 13 out of 20 participants with an initial incorrect test had obstruction and/or a low FVC; with similar findings in 11 out of 16 participants at week 28.

#### 6MWD

410 6MWTs were performed; 29 substudy participants did not perform a walk test at one or more time points, the main reasons being painful feet or rain. Median (IQR) 6MWD was 520 (465–576) m at week 0 and 585 (520–655) m at week 28 (table 2).

#### Chest radiograph score

Chest radiograph scores were available for 135 participants at week 0, and for 61 participants at week 28. Possible scores ranged from 0 to 140, with higher scores indicating more chest radiograph abnormalities. The median (IQR) chest radiograph scores were low: 4.0 (0.8–11.7) at week 0 and 0.9 (0–3.75) at week 28 (table 2). Cavities were present in eight (6%) participants at week 0 and one (2%) participant at week 28. Overall, chest radiograph scores showed significant correlation with respiratory symptoms, 6MWD and FEV_1_: for an increase of 10 points in chest radiograph score, an odds ratio of 1.51 for symptoms (95% CI 1.13–2.03, p=0.006) was observed (supplementary table S2), the average 6MWD decreased by 21 m (95% CI 11–31 m, p<0.001) and the average FEV_1_ decreased by 3.3% pred (95% CI 1.9–4.8% pred, p<0.001). However, in chest radiographs with lower scores (*i.e.* <10), the FEV_1_ varied markedly, and both normal and impaired lung function were seen in participants with little or no chest radiograph abnormalities (supplementary figure S2).

There was no significant association between chest radiograph score at week 0 and spirometry results at week 28 (odds ratio for normal lung function at week 28 per 10-point increase in chest radiograph score at week 0 was 0.77 (95% CI 0.55–1.08, p=0.13) and average FEV_1_ at week 28 was 1.87% pred lower (95% CI 3.98–−0.24% pred, p=0.08) for every 10-point increase of week 0 chest radiograph score).

### Effect of prednisone

**FIGURE 2:**
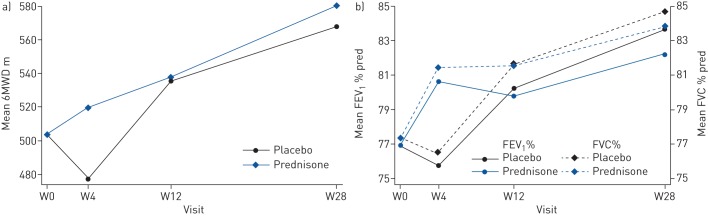
The effect of prednisone prophylaxis to prevent tuberculosis-associated immune reconstitution inflammatory syndrome (TB-IRIS) on lung function. Patients treated for HIV-associated TB received either prednisone or identical placebo during the first 4 weeks of antiretroviral therapy. Week (W)0 is the day when antiretroviral therapy and prednisone or placebo were started. a) Change over time of 6-min walk distance (6MWD) was statistically significantly associated with prednisone use (p=0.034); b) change over time of forced expiratory volume in 1 s (FEV_1_) and forced vital capacity (FVC) % predicted was statistically significantly associated with prednisone use (p=0.029 and p=0.015, respectively). Graphs represent data for nonsmokers. Curves for smokers are parallel.

There was no statistically significant difference in the change over time of symptoms (p=0.13) or chest radiograph score (p=0.92) between the prednisone and the placebo arm. The change in 6MWD over time was statistically significantly different between the groups (p=0.03), with the largest difference at week 4: participants in the prednisone arm walked 42 (95% CI 13–72) m further compared to participants in the placebo arm. In addition, change over time of both FEV_1_ and FVC were statistically significantly different between the two arms (p=0.03 and p=0.01), again most obvious at week 4, with those in the prednisone arm having FEV_1_ 4.9% pred (95% CI 0.7–9.0% pred) higher and FVC 4.9% pred (95% CI 1.3–8.5% pred) higher at week 4 compared to those in the placebo arm. Adjusting for the use of prednisone as treatment for TB-IRIS gave similar results (supplementary table S3). Baseline lung function did not statistically significantly affect the impact of prednisone (p=0.56 for FEV_1_) (supplementary table S4). At week 28, there was no longer a clear difference in either the 6MWD or FEV_1_ and FVC between the arms (figure 2 and table 3 and supplementary table S5).

**TABLE 3:** The effect of prednisone prophylaxis on change over time of pulmonary function parameters

	**Change over time of 6MWD m**	**p-value**	**Change in FEV_1_ % pred**	**p-value**
**Intercept average at week 0 for non-smokers**	504 (484–523)		504 (484–523)	
**Mean change**				
Effect of smoking (ever *versus* never)	14 (−11–39)	0.275	14 (−11–39)	0.275
**Mean change from week 0**				
Effect of time (visit)		<0.0001		<0.0001
Week 4	−27 (−50–−3)		−1.1 (−4.4–2.1)	
Week 12	32 (10–54)		3.3 (0.2–6.4)	
Week 28	64 (42–86)		6.8 (3.6–10.0)	
Effect of prophylactic prednisone		0.034		0.029
Week 4	42 (13–72)		4.9 (0.7–9.0)	
Week 12	2 (−26–30)		−0.4 (−4.4–3.5)	
Week 28	13 (−15–41)		−1.5 (−5.6–2.6)	

Data are presented as intercept and estimated coefficients (95% CI) from the mixed-effects regression models, unless otherwise stated. Data are adjusted for all other covariates presented in the table. Effect of time (visit) refers to the effect of time in the placebo arm. Because allocation to either the prednisone or the placebo arm was randomised, no adjustment for baseline variables other than smoking was made. 6MWD: 6-min walk distance; FEV_1_: forced expiratory volume in 1 s.

### Effect of TB-IRIS

**FIGURE 3:**
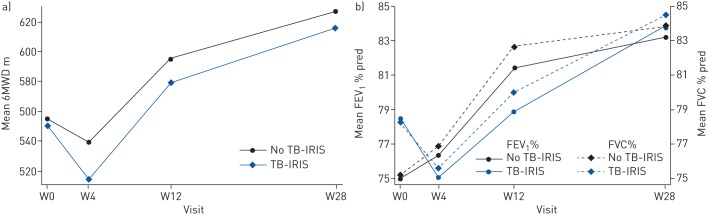
The effect of the development of tuberculosis-associated immune reconstitution inflammatory syndrome (TB-IRIS) on lung function. Patients with TB-IRIS are compared to those without TB-IRIS. a) Change over time of 6-min walk distance (6MWD) was not statistically significantly associated with TB-IRIS (p=0.62); b) change over time of forced expiratory volume in 1 s (FEV_1_) and forced vital capacity (FVC) % predicted was not statistically significantly associated with TB-IRIS (p=0.11 and p=0.054, respectively). Graphs represent data for male nonsmokers in the placebo arm aged 40 years with pulmonary TB, an HIV viral load at screening of 800 000 copies·mL^−1^, a CD4 at screening of 100 cells·μL^−1^ and did not have previous TB. The differences between TB-IRIS and no TB-IRIS were similar for other patient profiles. W: week.

When comparing participants who developed paradoxical TB-IRIS to those who did not, TB-IRIS was associated with a change in the presence of symptoms over time (p=0.03), but there was no statistically significant difference in change over time of FEV_1_ % pred (p=0.11), FVC % pred (p=0.054), 6MWD (p=0.62) or chest radiograph score (p=0.20) (figure 3, table 4 and supplementary table S6).

**TABLE 4:** The effect of tuberculosis-associated immune reconstitution inflammatory syndrome (TB-IRIS) on change over time of pulmonary function parameters

	**6MWD m**	**p-value**	**Mean change in FEV_1_% pred**	**p-value**
**Change over time**				
Intercept (average if all other covariates are 0)	837 (711–964)		83.9 (57.5–110.3)	
Mean change				
Effect of TB-IRIS at week 0	−4 (−35–27)	0.79	3.2 (−2.7–9.2)	0.29
Effect of smoking (ever *versus* never)	−44 (−69–−19)	0.001	−5.9 (−10.6–−1.1)	0.02
Effect of age (per increase of 1 year in age at week 0)	−3 (−4–−2)	<0.001	0.04 (−0.2–0.3)	0.76
Effect of sex (female *versus* male)	−112 (−138–−86)	<0.001	−3.6 (−9.0–1.8)	0.19
Effect of type of TB (participants without signs of extrapulmonary TB *versus* those with signs of extrapulmonary TB)	30 (−14–74)	0.19	0.7 (−8.6–10.1)	0.88
Effect of HIV viral load (per log_10_ cps·mL^−1^ increase at screening)	−32 (−50–−13)	0.001	−0.5 (−4.5–3.4)	0.79
Effect of CD4 count (per increase of 10 CD4 cells·μL^−1^ at screening)	−2 (−4–1)	0.21	−0.8 (−1.3–−0.2)	0.005
Effect of previous tuberculosis	−21 (−60–18)	0.28	−14.8 (−23.4–−6.1)	0.001
**Mean change week 0**				
Effect of time (visit)		<0.0001		<0.001
Week 4	−16 (−45–14)		1.4 (−3.0–5.7)	
Week 12	40 (13–67)		6.3 (2.3–10.4)	
Week 28	72 (44–100)		8.1 (3.9–12.3)	
Effect of TB-IRIS		0.68		0.06
Week 4	−21 (−54–13)		−4.7 (−9.4–−0.1)	
Week 12	−11 (−44–21)		−6.0 (−10.5–−1.4)	
Week 28	−7 (−40–27)		−2.8 (−7.6–2.0)	
Effect of prophylactic prednisone		0.036		0.07
Week 4	41 (13–70)		4.4 (0.2–8.7	
Week 12	0 (−27–26)		−0.1 (−4.8– 3.2)	
Week 28	4 (−22–31)		−1.3 (−5.5–2.8)	

Data are presented as intercept and estimated coefficients (95% CI) from the mixed-effects regression models, unless otherwise stated. Data are adjusted for all other covariates presented in the table. 6MWD: 6-min walk distance; FEV_1_: forced expiratory volume in 1 s.

16 participants developed TB-IRIS without any respiratory signs or symptoms. Exclusion of these nonpulmonary IRIS cases from the analysis did not affect the results (data not shown).

## Discussion

We assessed pulmonary function in a cohort of patients with HIV-associated TB at high risk of TB-IRIS, enrolled in a trial investigating the efficacy of prophylactic prednisone in preventing TB-IRIS.

Respiratory symptoms were common early during TB treatment and abnormal spirometry (low FVC and airflow obstruction with and without low FVC) was found in 66% of participants with acceptable spirometry. At the end of TB treatment, symptoms persisted in 8% and abnormal spirometry in 50% of participants. The proportion of abnormal spirometry results is higher than expected for either the general or HIV-infected population [1, 34], but is comparable to results from other studies in HIV-associated TB patients [4, 5, 19].

In our trial, open-label treatment of TB-IRIS with prednisone was allowed. This resulted in 30 out of 76 participants in the placebo arm receiving prednisone for the treatment of TB-IRIS and the majority of the participants who developed TB-IRIS receiving prednisone, either as prophylaxis, as treatment, or both, with prednisone treatment given to the more severe cases of TB-IRIS. This limits our evaluation of the individual effects of both prednisone as well as TB-IRIS on lung function.

Within these limitations, we did not find an effect of TB-IRIS on spirometric lung function over time. This contradicts findings of two recent studies [18, 19] that hypothesised that TB-IRIS-like increases in inflammation may lead to decreased lung function, which may occur in patients with HIV-associated TB initiating ART, even in the absence of clinically overt TB-IRIS. It is possible that in the present study, mild TB-IRIS did not result in sufficient additional pulmonary inflammation to affect long-term respiratory outcomes, whereas in severe TB-IRIS the effect was ameliorated by treatment with prednisone.

We found prophylactic prednisone affected change over time of 6MWD and FEV_1_ and FVC, primarily at week 4, when participants completed their study prednisone, potentially by preventing TB-IRIS. Consequently, the higher proportion of participants with TB-IRIS in the placebo arm may be responsible for the demonstrated favourable effect of prophylactic prednisone on lung function. Additionally, prednisone can directly improve exercise performance [35] and FEV_1_ in other disease processes, for example acute exacerbations of chronic obstructive pulmonary disease [36]. Ravimohan *et al.* [18] found that decreased lung function after 4 weeks of TB treatment impacts negatively on long-term lung function, and Auld *et al.* [19] found similar associations, but only in severe lung function declines. In the current study, despite an increase in lung function at week 4, we did not observe any long-term effects of prophylactic prednisone on lung function. This could be due to Type 2 error, with a large proportion of participants in the placebo arm receiving prednisone as treatment for TB-IRIS. Alternatively, prednisone may have been given too late in the disease process and prescribed only after lung damage had already occurred.

We found a high proportion of participants with technically incorrect spirometry results early on during treatment: a finding not reported previously. In studies performed in participants without TB, in the latter stages of TB treatment or after TB treatment, 2–30% did not perform spirometry correctly and these patients were subsequently excluded [1, 3–5, 9, 18, 37]. We considered that the participants who did not perform spirometry correctly might include those with worse pulmonary status: for example, in severely ill patients, and in those with significant cough and/or shortness of breath spirometry is technically challenging. This hypothesis was supported in our study by finding that the majority of technically incorrect spirometric results were due to inability of patients to exhale for 6 s. Furthermore, the association with a shorter 6MWD and a lower KPS, and abnormal lung function in the majority of these participants at follow-up spirometry testing adds weight to this argument. Thus, excluding these participants from the analyses may underestimate the burden of lung function impairment in TB. In our study, when technically incorrect results were included as abnormal, the percentage abnormal tests at baseline increased from 66% to 74%.

In keeping with published data on HIV-associated TB patients with a low CD4 count [12], only a small proportion of participants demonstrated extensive chest radiograph abnormalities, while cavitation was uncommon. Our finding of a negative association between chest radiograph score and FEV_1_, although statistically significant, needs to be interpreted with caution. Although a high score is unsurprisingly associated with lower FEV_1_, a low score does not appear to rule out significant abnormality in FEV_1_. Several other studies, using many different scoring methods, have reported the relationship between FEV_1_ and chest radiograph score in TB [3, 5, 10, 38, 39]. However, all studies describe more severe chest radiograph abnormalities than our present study, and few included HIV-positive patients. Those that did chose not to report data on HIV patients specifically, or claimed that the numbers were too small for meaningful subanalysis. Our observation that the presence of a normal chest radiograph in HIV-associated TB patients with a CD4 count ≤100 cells·μL^−1^ does not exclude lung function abnormalities, is probably explained by the insensitivity of chest radiograph to detect changes responsible for the reduced FEV_1_, for example small airways and subtle parenchymal abnormalities.

Besides the considerable overlap between participants developing TB-IRIS and participants prescribed prednisone to treat TB-IRIS, our study has other limitations. First, as consequence of the substudy commencing after the main trial, we do not have complete data on all participants. Second, we do not have reliable information about the time between the start of symptoms and the start of TB treatment, with longer duration of symptoms being a risk factor for pulmonary impairment [5, 40]. Third, normal values for 6MWD in our population are lacking. Most participants walked relatively far, possibly because of the relatively short duration of illness and possibly younger age of participants when compared with other chronic lung diseases. Finally, the use of the NHANES reference range (derived in North American populations) may have resulted in an overestimate of lung function impairment, as normal values for FEV_1_ and FVC tend to be higher than those for African populations [41].

In conclusion, we found that lung function impairment is common in patients with HIV-associated TB. Prednisone to prevent TB-IRIS improved lung function at week 4, possibly by reducing TB-IRIS; however, the 28-day course of prednisone did not improve lung function from week 12 onwards in patients with CD4 counts ≤100 cells·μL^−1^. Overlap between the groups through the development of TB-IRIS and subsequent use of prednisone as treatment limits our ability to make definitive conclusions. Prednisone remains recommended to prevent TB-IRIS in this population based on the findings of the main PredART trial [28], despite this study being unable to demonstrate long-term benefits in lung function. Further studies, using PET-CT imaging and other biomarkers of inflammation and lung damage in TB [13] are needed to better understand the pathogenesis of lung function impairment in HIV-associated TB.

## Supplementary material

10.1183/13993003.01692-2019.Supp1 **Please note:** supplementary material is not edited by the Editorial Office, and is uploaded as it has been supplied by the author.
Supplementary material ERJ-01692-2019.SUPPLEMENT

## Shareable PDF

10.1183/13993003.01692-2019.Shareable1 This one-page PDF can be shared freely online.
Shareable PDF ERJ-01692-2019.Shareable
