# Supplementary material for: The effect of HIV-associated tuberculosis, tuberculosis-IRIS and prednisone on lung function
Source: Eur Respir J. 2020 Mar 12;55(3):1901692. doi: 10.1183/13993003.01692-2019 (PMC7066470; doi:10.1183/13993003.01692-2019)
Supplement: Supplementary file 1 [file ERJ-01692-2019.SUPPLEMENT.pdf]

The effect of HIV-associated tuberculosis, tuberculosis-IRIS and prednisone on lung function

Cari Stek, Brian Allwood, Elsa du Bruyn, Jozefien Buyze, Charlotte Schutz, Friedrich Thienemann, Adele Lombard, Robert J. Wilkinson, Graeme Meintjes and Lutgarde Lynen

Online supplement

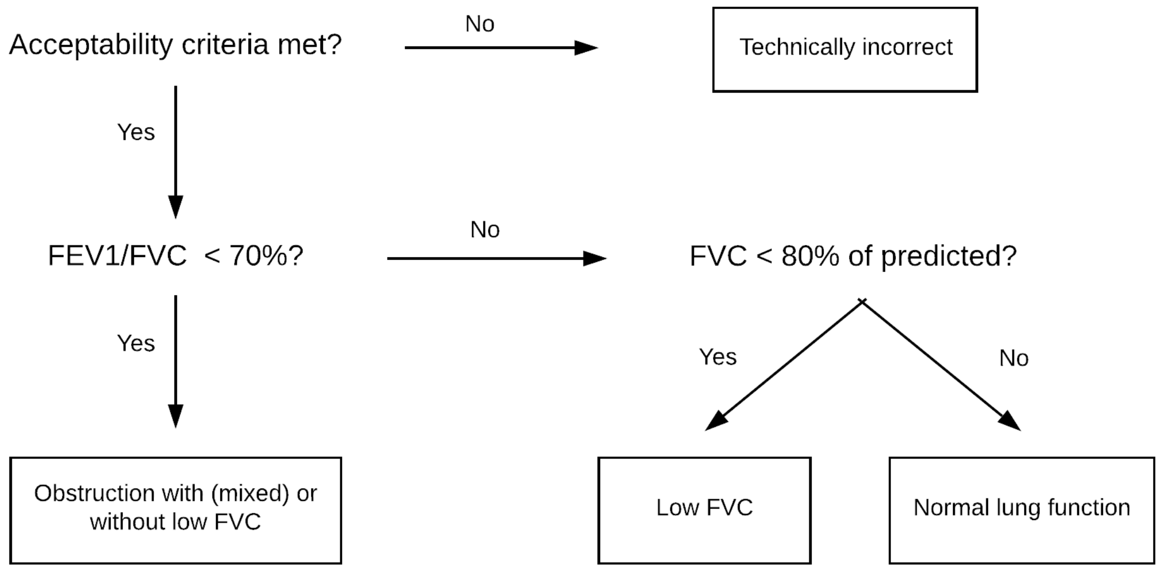

Figure S1 Definitions of different spirometry outcomes

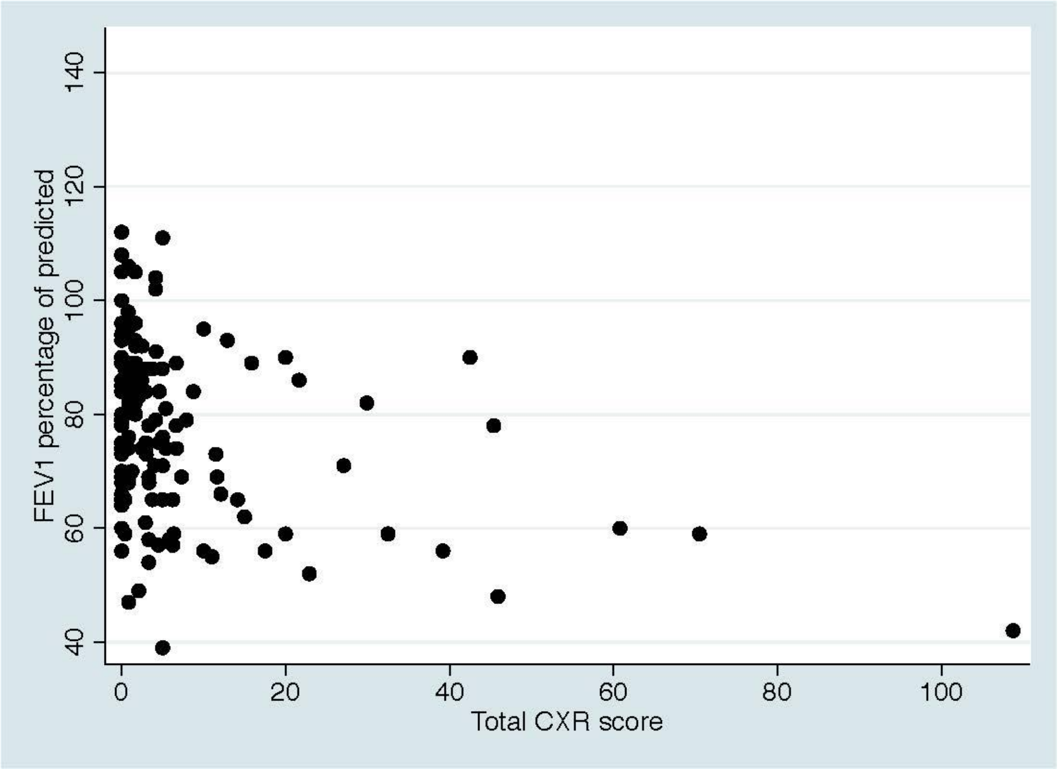

Figure S2 The association between chest X-ray score and FEV1 percentage of predicted (n = 135). Chest X-rays were scored using an adapted version of the Timika score[1)]: visible lung fields on the chest X-ray were divided into six zones and the percentage of affected lung in each zone and its prominent opacification type were estimated. A total score was generated by adding up the percentages of each zone and dividing the total by the number of scored zones (usually 6), adding an additional 40 points if one or more cavities > 1cm in diameter were present. Therefore, total scores could range from 0 to 140. Chest X-ray scores were statistically significantly correlated with FEV1 percentage of predicted ($p < 0.001$).

[1)] Kriel M, Lotz JW, Kidd M, Walzl G. Evaluation of a radiological severity score to predict treatment outcome in adults with pulmonary tuberculosis. Int J Tuberc Lung Dis 2015: 19(11): 1354-1360

TABLE S1 Acceptability criteria for the recording of FVC and FEV1 using spirometry

**No artefacts**	Coughing during first second of expiration<br>Glottis closure<br>Early termination or submaximal effort<br>Leak<br>Obstructed mouthpiece
**Good starts**	Extrapolated volume < 5% of FVC or 0,15l, whichever is greater
**Exhalation**	Duration ≥ 6 s, plateau in the volume-time curve, or if the subject cannot or should not continue

Miller MR, Hankinson J, Brusasco V, Burgos F, Casaburi R, Coates A, Crapo R, Enright P, van der Grinten CP, Gustafsson P, Jensen R, Johnson DC, MacIntyre N, McKay R, Navajas D, Pedersen OF, Pellegrino R, Viegi G, Wanger J, Force AET. Standardisation of spirometry. *Eur Respir J* 2005; 26: 319-338

TABLE S2 Association between chest X-ray score and respiratory symptoms

	Odds ratio	p-value
Cough	1.24	0.08
Dyspnoea at exertion	1.45	0.007
Dyspnoea at rest	1.03	0.89
Total	1.51	0.006

Total = cough, and/or dyspnea at exertion, and/or dyspnea at rest. Odds ratio's are calculated for an increase of 10 units in chest-X-ray score. A p-value of < 0.05 is considered significant.

TABLE S3 The effect of prednisone prophylaxis on change over time of pulmonary function parameters, adjusted for prednisone as treatment

Change over time of six-minute walking distance (6MWD) in meters

	6MWD	95% CI	p-value
**Intercept**<br>(average 6MWD if all other co-variates are 0)	662	595 – 729	

	Mean change in 6MWD	95% CI	p-value
Effect of prednisone as treatment	-20	-44 - 4	0.097
Effect of smoking (ever vs never)	-45	-70 - -20	<0.001
Effect of age (per increase of one year in age at week 0)	-3	-4 – -1	<0.001
Effect of gender (female vs male)	-108	-133 – -82	<0.001
Effect of type of TB (participants without signs of extrapulmonary TB vs those with signs of extrapulmonary TB)	35	-9 – 78	0.12
Effect of HIV viral load (per log_{10} cps/ml increase at screening)	-1	-2 – -1	<0.001
Effect of CD4 count (per increase of 10 CD4 cells/µl at screening)	-1	-3 – 2	0.48
Effect of previous tuberculosis	-26	-64 – 13	0.19

	Mean change in 6MWD from week 0	95% CI	p-value
**Effect of time (visit)**			<0.0001
week 4	-27	-50 – -4	
week 12	34	13 – 56	
week 28	69	48 – 91	
**Effect of prophylactic prednisone**			0.020
week 4	44	15 – 72	
week 12	-0.03	-27 – 27	
week 28	3	-23 – 30	

Change over time of forced expiratory volume in 1 second (FEV1) as % of predicted value

	FEV1 %	95% CI	p-value
**Intercept**<br>(average FEV1 % if all other co-variates are 0)	80.7	65.9 – 95.5	

	Mean change in FEV1 %	95% CI	p-value
Effect of prednisone as treatment	1.5	-3.7 – 6.8	0.57
Effect of smoking (ever vs never)	-5.6	-10.3 - -0.8	0.022
Effect of age (per increase of one year in age at week 0)	0.06	-0.2 – 0.3	0.66
Effect of gender (female vs male)	-3.5	-8.8 – 1.9	0.21
Effect of type of TB (participants without signs of extrapulmonary TB vs those with signs of extrapulmonary TB)	0.7	-8.6 – 10.1	0.88
Effect of HIV viral load (per log_{10} cps/ml increase at	0.04	-0.1 – 0.2	0.64

screening)			
Effect of CD4 count (per increase of 10 CD4 cells/µl at screening)	-0.7	-1.3 – -0.2	0.008
Effect of previous tuberculosis	-14.8	-23.4 – -6.2	0.001

	**Mean change in FEV1 % from week 0**	**95% CI**	**p-value**
**Effect of time (visit)**			<0.0001
week 4	-1.5	-4.8 – 1.8	
week 12	2.9	-0.3 – 6.1	
week 28	6.4	3.1 – 9.6	
**Effect of prophylactic prednisone**			0.043
week 4	5.1	0.9 – 9.4	
week 12	-0.04	-4.1 – 4.0	
week 28	-1.0	-5.1 – 3.2	

Intercept and estimated coefficients with their 95% confidence intervals (95% CI) from the mixed effects regression models are listed. Data are adjusted for all other covariates presented in the table. We have not adjusted for TB-IRIS because we assume it to be on the causal pathway.

TABLE S4 The effect of baseline spirometry outcome on the effect of prednisone on change over time of forced expiratory volume in 1 second (FEV1) as % of predicted value

	FEV1 %	95% CI	
**Intercept**			
(average FEV1 % at week 0 for non-smokers with abnormal spirometry result)	67.1	63.4 - 70.8	<0.001

	Mean change in FEV1 %	95% CI	p-value
Effect of spirometry outcome at baseline			
(normal vs abnormal)	25.6	20.2 - 31.0	<0.001
Effect of smoking (ever vs never)	-2.0	-6.0 – 2.0	0.33

	Mean change in FEV1 % from week 0	95% CI	p-value
**Effect of time (visit)**			<0.0001
week 4	-0.3	-4.5 – 3.8	
week 12	4.3	0.4 – 8.2	
week 28	9.8	5.6 – 14.0	
**Effect of prophylactic prednisone**			0.12
week 4	5.9	0.7 – 11.2	
week 12	0.8	-4.5 – 6.0	
week 28	-0.4	-6.1 – 5.3	
**Effect of normal spirometry at baseline**			0.41
week 4	-0.4	-7.6 – 6.7	
week 12	-3.0	-10.5 – 4.5	
week 28	-6.7	-15.0 – 1.6	
**Effect of normal spirometry at baseline on the effect of prophylactic prednisone**			0.56
week 4	-5.3	-14.2 – 3.6	
week 12	-5.7	-15.0 – 3.7	
week 28	-3.3	-13.6 – 7.1	

Intercept and estimated coefficients with their 95% confidence intervals (95% CI) from the mixed effects regression models are listed. Data are adjusted for all other covariates presented in the table. Because allocation to either the prednisone or the placebo arm was randomized, no adjustment for baseline variables other than smoking and baseline spirometry outcome was done. Only participants with who had a baseline spirometry test done (n = 83) were included in this analysis.

TABLE S5 The effect of prednisone prophylaxis on change over time of forced vital capacity (FVC) as % of predicted value

	FVC %	95% CI	
**Intercept**			
(average FVC % at week 0 for non-smokers)	77.3	74.4 – 80.3	
	**Mean change in FVC %**	**95% CI**	**p-value**
**Effect of smoking (ever vs never)**	-5.0	-8.7 – -1.2	0.009
	**Mean change in FVC % from week 0**	**95% CI**	**p-value**
**Effect of time (visit)**			<0.0001
week 4	-0.8	-3.7 – 2.0	
week 12	4.3	1.6 – 7.0	
week 28	7.3	4.5 – 10.1	
**Effect of prophylactic prednisone**			0.015
week 4	4.9	1.3 – 8.5	
week 12	-0.1	-3.5 – 3.4	
week 28	-0.8	-4.4 –2.7	

Intercept and estimated coefficients with their 95% confidence intervals (95% CI) from the mixed effects regression models are listed. Data are adjusted for all other covariates presented in the table.

TABLE S6 The effect of tuberculosis-associated immune reconstitution inflammatory syndrome (TB-IRIS) on change over time of forced vital capacity (FVC) as % of predicted value

	FVC %	95% CI	p-value
**Intercept**			
(average FVC % if all other co-variates are 0)	80.3	55.7 – 104.8	<0.001

	Mean change in FVC %	95% CI	p-value
Effect of TB-IRIS at week 0	2.9	-2.6 – 8.3	0.30
Effect of smoking (ever vs never)	-5.9	-10.1 – -1.6	0.007
Effect of age (per increase of one year in age at week 0)	0.1	-0.2 – 0.4	0.45
Effect of gender (female vs male)	-2.7	-7.8 – 2.3	0.29
Effect of type of TB (participants without signs of extrapulmonary TB vs those with signs of extrapulmonary TB)	3.7	-5.0 – 12.5	0.40
Effect of HIV viral load (per log_{10} cps/ml increase at screening)	-1.0	-4.6 – 2.7	0.61
Effect of CD4 count (per increase of 10 CD4 cells/µl at screening)	-0.7	-1.2 – -0.2	0.009
Effect of previous tuberculosis	-10.6	-18.7 – -2.6	0.010

	Mean change in FVC % from week 0	95% CI	p-value
**Effect of time (visit)**			<0.0001
week 4	1.7	-2.0 – 5.5	
week 12	7.5	3.9 – 11.0	
week 28	8.7	5.0 – 12.3	
**Effect of TB-IRIS**			0.026
week 4	-4.4	-8.5 – -0.4	
week 12	-5.7	-9.7 – -1.8	
week 28	-2.5	-6.7 – 1.7	
**Effect of prophylactic prednisone**			0.040
week 4	4.5	0.7 – 8.2	
week 12	-0.4	-4.0 – 3.1	
week 28	-0.8	-4.4 – 2.9	

Intercept and estimated coefficients with their 95% confidence intervals (95% CI) from the mixed effects regression models are listed. Data are adjusted for all other covariates presented in the table
