# Supplementary material for: The effect of HIV-associated tuberculosis, tuberculosis-IRIS and prednisone on lung function
Source: Eur Respir J. 2020 Mar 12;55(3):1901692. doi: 10.1183/13993003.01692-2019 (PMC7066470; doi:10.1183/13993003.01692-2019)
Supplement: Supplementary file 2 [file ERJ-01692-2019.Shareable.pdf]

SHAREABLE PDF

The effect of HIV-associated tuberculosis, tuberculosis-IRIS and prednisone on lung function

Cari Stek[1,2,3], Brian Allwood[4], Elsa Du Bruyn[2,3], Jozefien Buyze[1], Charlotte Schutz[2,3], Friedrich Thienemann[2,3], Adele Lombard[4], Robert J. Wilkinson[2,3,5,6], Graeme Meintjes[2,3] and Lutgarde Lynen[1]

Affiliations: [1]Dept of Clinical Sciences, Institute of Tropical Medicine, Antwerp, Belgium. [2]Wellcome Centre for Infectious Diseases Research in Africa, Institute of Infectious Disease and Molecular Medicine, University of Cape Town, Cape Town, South Africa. [3]Dept of Medicine, University of Cape Town, Cape Town, South Africa. [4]Division of Pulmonology, Dept of Medicine, Stellenbosch University, Stellenbosch, South Africa. [5]Dept of Medicine, Imperial College London, London, UK. [6]The Francis Crick Institute, London, UK.

Correspondence: Cari Stek, Wellcome Centre for Infectious Diseases Research in Africa, Institute of Infectious Disease and Molecular Medicine, Faculty of Health Sciences, University of Cape Town, Anzio Rd, Observatory 7925, Cape Town, South Africa. E-mail: cari_stek@hotmail.com

@ERSpublications
Post-tuberculosis lung disease is common in patients with HIV-associated TB at high risk of TB-IRIS (CD4 count ⩽100 cells·µL^{-1}). Neither TB-IRIS itself, nor prednisone treatment, affected long-term pulmonary outcomes in a South African clinical setting. http://bit.ly/2RjMl9c

Cite this article as: Stek C, Allwood B, Du Bruyn E, *et al.* The effect of HIV-associated tuberculosis, tuberculosis-IRIS and prednisone on lung function. *Eur Respir J* 2020; 55: 1901692 [https://doi.org/10.1183/13993003.01692-2019].

This single-page version can be shared freely online.

ABSTRACT Residual pulmonary impairment is common after treatment for tuberculosis (TB). Lung function data in patients with HIV-associated TB are scarce, especially in the context of paradoxical TB-associated immune reconstitution inflammatory syndrome (TB-IRIS) and prophylactic prednisone. We aimed to determine the prevalence of lung function abnormalities in patients with HIV-associated TB and CD4 counts ⩽100 cells·µL^{-1} and assess the effect of prophylactic prednisone and the development of paradoxical TB-IRIS on pulmonary impairment.

We performed spirometry, 6-min walk test (6MWT) and chest radiography at baseline (week 0) and at weeks 4, 12 and 28 in participants of the PredART trial, which evaluated a 28-day course of prednisone to prevent TB-IRIS in patients with HIV-associated TB commencing antiretroviral therapy.

153 participants underwent spirometry and/or 6MWT at one or more time points. Abnormal spirometry measurements were present in 66% of participants at week 0 and 50% at week 28; low forced vital capacity was the commonest abnormality. Chest radiographs showed little or no abnormalities in the majority of participants. Prednisone use resulted in a 42 m greater 6-min walk distance and a 4.9% higher percentage of predicted forced expiratory volume in 1 s at week 4; these differences were no longer significantly different from week 12 onwards. TB-IRIS did not significantly impair lung function outcome.

Residual pulmonary impairment is common in HIV-associated TB. In patients with low CD4 counts, neither prophylactic prednisone as used in our study nor the development of TB-IRIS significantly affected week-28 pulmonary outcome.

Copyright ©ERS 2020. This version is distributed under the terms of the Creative Commons Attribution Licence 4.0.
